# Clinical features and management of closed injury of the cervical trachea due to blunt trauma

**DOI:** 10.1186/1757-7241-21-60

**Published:** 2013-08-07

**Authors:** Dong Ye, Zhisen Shen, Yuyuan Zhang, Shijie Qiu, Cheng Kang

**Affiliations:** 1 Department of Otorhinolaryngology-Head and Neck Surgery, Lihuili Hospital of Ningbo University, Ningbo 315040, China; 2 Ningbo University School of Medicine, Ningbo 315211, China

**Keywords:** Tracheal disruption, Diagnosis, Management, Tracheotomy, Repair

## Abstract

**Background:**

We retrospectively reviewed the presentation, diagnosis, treatment, and outcomes of patients with closed injury of the cervical trachea. We evaluated factors that improve diagnosis and treatment, reduce mortality, and avoid tracheal stenosis.

**Methods:**

We reviewed the clinical data of 17 patients with closed injury of the cervical trachea. All patients underwent CT scanning or endoscopy, tracheal exploration, low tracheotomy, and tracheal repair.

**Results:**

In 12 patients, breathing, phonation, and swallowing functions had returned to normal at 2 weeks. In three patients, breathing and swallowing functions had recovered at 2 weeks, but hoarseness continued. In two patients, tracheal stenosis prevented extubation and required further surgery; in these patients breathing and swallowing functions had recovered at 6 months.

**Conclusions:**

Closed injury of the cervical trachea may cause airway obstruction and is potentially life-threatening. Early diagnosis and repair to restore structure and function are important to ensure survival and avoid tracheal stenosis.

## Background

Closed injury to the cervical trachea results from a violent impact to the neck, which pushes the trachea against the rigid cervical spine, causing tracheal cartilage and soft tissue lacerations but leaving the skin intact. This injury is rare because of the flexibility of the tracheal cartilages and the protection offered by the mandible, manubrium of the sternum, clavicles, and cervical spine. The cervical trachea is located deep in the neck, and is closely associated with the major cervical vessels, nerves, larynx, and esophagus, which are also frequently injured in patients with tracheal injury. This rare and serious injury is challenging to manage, even for doctors with experience in managing difficult airways. The lack of a skin wound may delay diagnosis. The complex clinical manifestations and rapid progression of severity are often life-threatening. Early diagnosis and treatment are therefore important to reduce severe morbidity and mortality [1]. We reviewed the records of 17 patients who were treated for closed injury of the cervical trachea due to blunt trauma in the Department of Otorhinolaryngology–Head and Neck Surgery, Lihuili Hospital of Ningbo University, from January 2000 to May 2012. We recorded the mechanisms of injury, clinical features, diagnostic methods, early airway management, definitive treatment, and outcomes of these patients by retrospective review of the case notes, and evaluated the anonymized data. The study protocol conforms to the ethical standards of the Declaration of Helsinki.

## Methods

### General information

We identified 17 patients who were eligible for inclusion in the study, including 14 males and three females with a mean age of 41 years (range 19–50 years). The causes of injury included traffic accidents (six cases), clothesline type injuries from impact with a rope (three cases), impact with a pipe (two cases), and one case each of impact with the handlebars of a motorcycle during emergency braking, impact with the steering wheel of a car while driving, injury caused by a workplace machine, fall, impact with a hoe handle, and impact with a steel plate. The trachea was completely ruptured in 11 patients and partially ruptured in six patients.

### Clinical manifestations and examination

**Figure 1:**
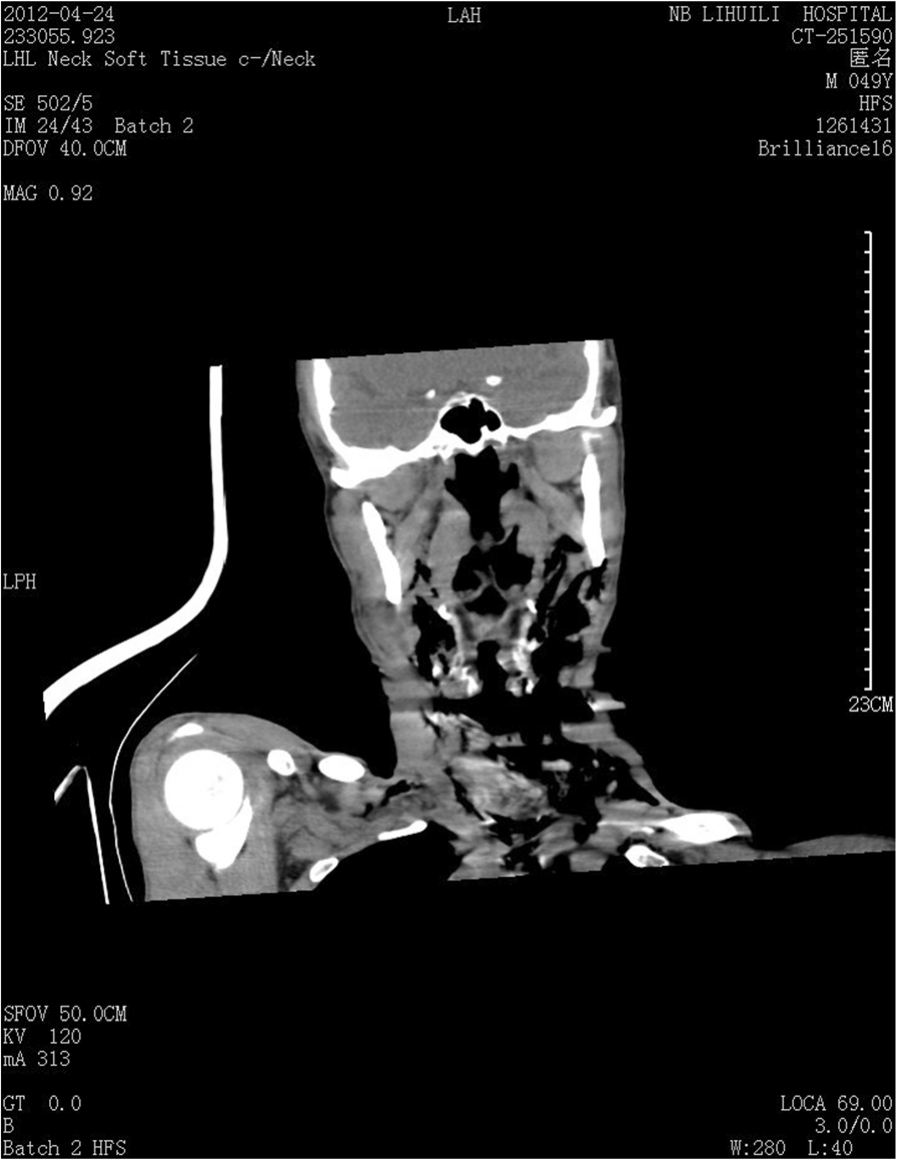
CT scan of the neck, showing disruption of the cervical trachea and widespread subcutaneous emphysema.

**Figure 2:**
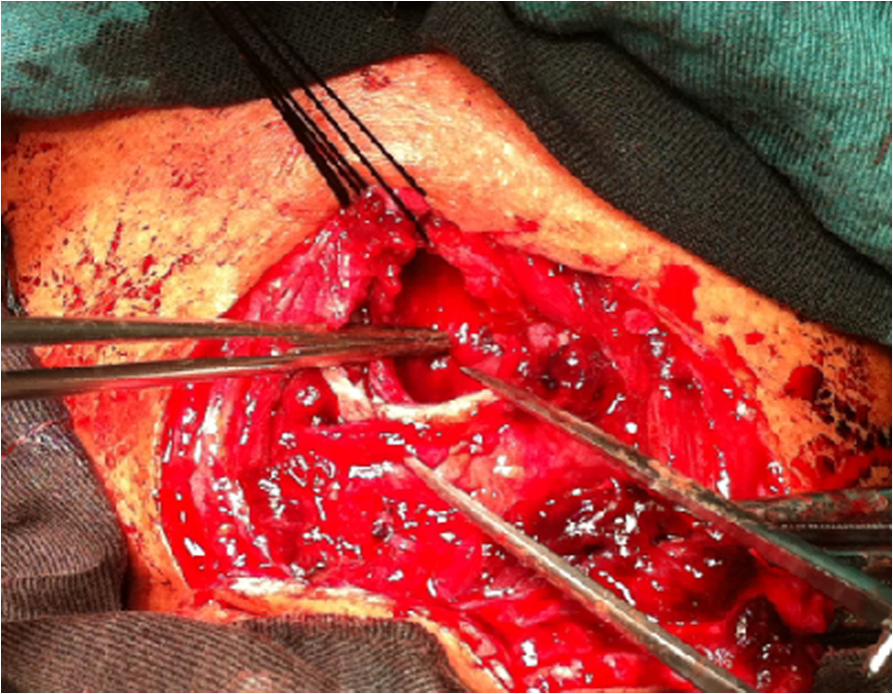
Intraoperative view, showing complete disruption of the cervical trachea and severe damage to the surrounding tissues.

**Figure 3:**
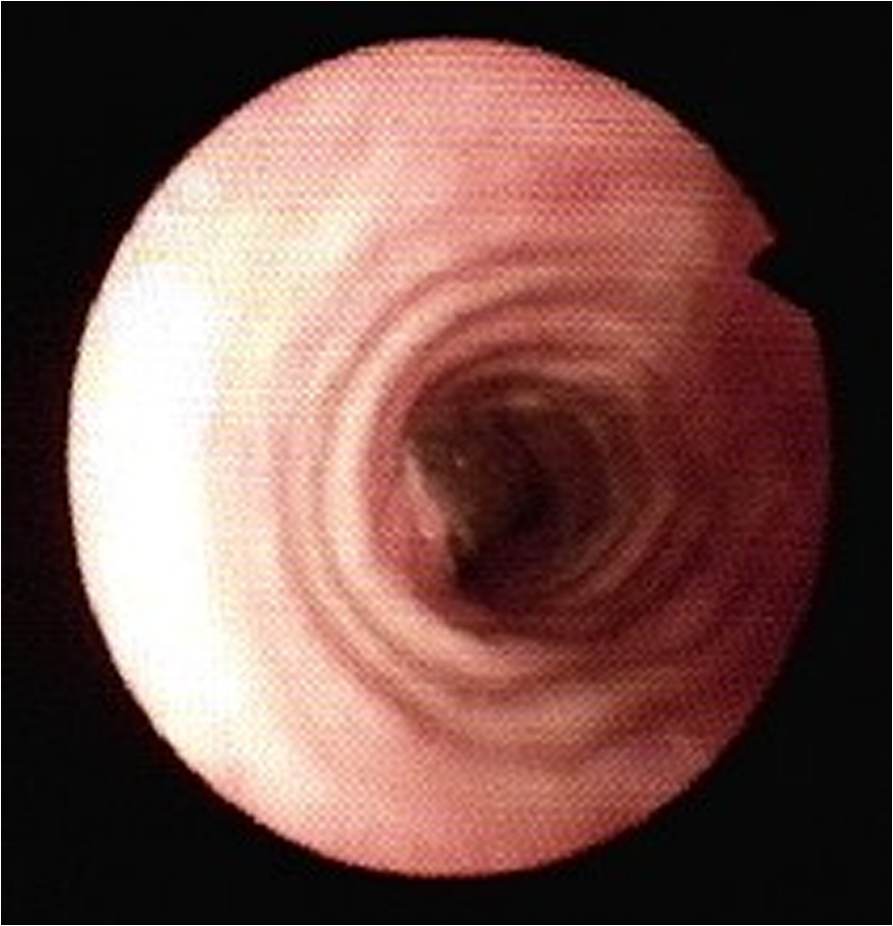
**Fiberoptic laryngoscopy image at 16 days after surgery.** No anastomotic stricture is seen.

**Figure 4:**
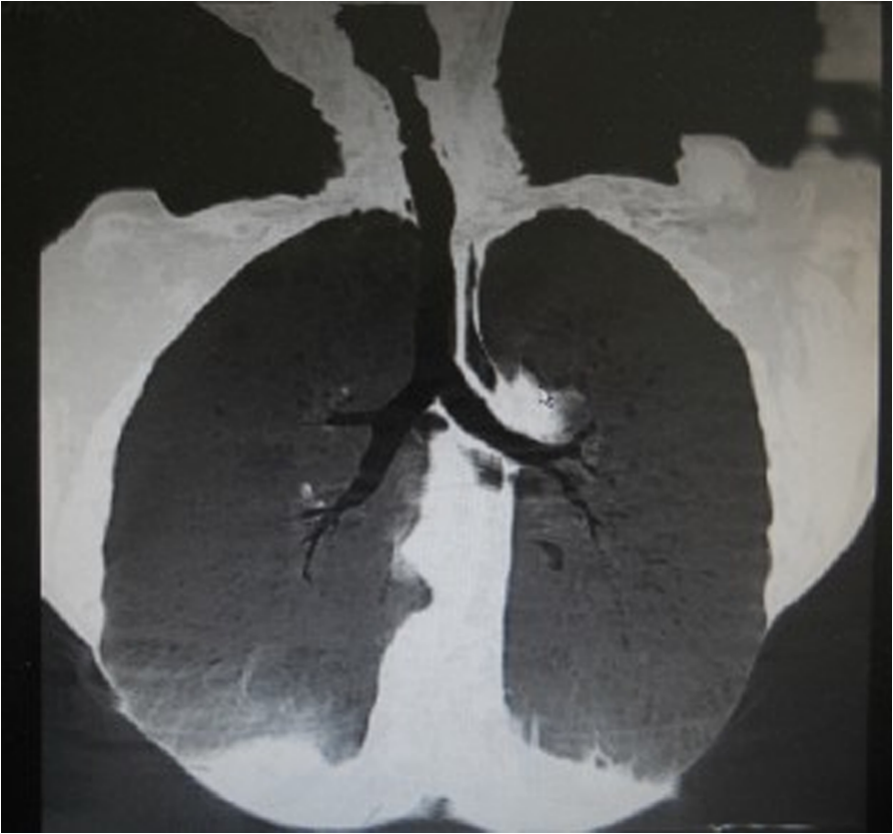
**CT scan of the neck at 2 months after surgery.** The tracheal injury has healed well with no proliferation of granulation tissue.

The main symptoms at presentation were neck pain when swallowing, varying degrees of dyspnea, coughing, bloodstained saliva or hemoptysis, hoarseness, and dysphagia. The main findings on physical examination were cyanosis, pneumothorax, vocal cord paralysis, aphonia, and extensive subcutaneous emphysema and bruising with rapid expansion to the neck, shoulder, and chest. Seven patients had significant associated injuries such as blunt laryngeal trauma, disruption of the larynx and tracheal rings, and tracheal shift or deformation. One patient had bilateral pneumothorax, one had a cerebral injury, one had a cervical spine injury, one had a mandibular fracture, one had an esophageal injury, and one had a rib fracture. All patients underwent neck and chest X-rays, CT scans, or endoscopy; and surgical exploration (Figures 1, 2, 3, and 4).

### Treatment

All patients were assessed for neck trauma, and received fluid resuscitation to treat shock including blood transfusion if needed. Four patients with grade III or IV dyspnea underwent tracheotomy before arrival at the hospital. Some patients underwent anterior midline neck incision under local anesthesia with separation of the anterior neck muscles, exploration of the tracheal injury, and suctioning of the trachea. The trachea was then intubated through the site of injury under general anesthesia. If the distal end of the trachea had retracted into the chest, it was pulled back up and intubated, and the end was trimmed. If the tracheal cartilages were severely damaged, one or two rings were resected and the ends were anastomosed back together using 3–0 absorbable sutures spaced 2.5 mm apart. Repair of the larynx, thyroid, and other important adjacent tissues was performed if needed. If both the trachea and esophagus were seriously injured, they were repaired and then reinforced using the surrounding tissues. If the tracheal cartilages were severely damaged for a length of up to 5 cm, some broken cartilage was removed followed by end-to-end anastomosis of the tracheal ends. If the tracheal cartilages were damaged over more than 5 cm, a nitinol stent or silicone tube was used to prevent tracheal stenosis, which was removed after 10–14 days. Seven patients with laryngeal trauma underwent open laryngeal exploration and laryngotracheal plasty. In one case, the recurrent laryngeal nerves were injured bilaterally. This patient underwent secondary recurrent laryngeal nerve to phrenic nerve anastomosis, and implantation of the end of the recurrent laryngeal nerve in the adductor muscle into the posterior cricoarytenoid muscle. One patient had disruption of the left recurrent laryngeal nerve only. One patient had left recurrent laryngeal nerve dysfunction due to severe edema, but did not receive specific treatment for this. All patients underwent debridement, repair of injuries, and layered closure of incisions. In the patient with a mandibular fracture, the fracture was fixed at the same time as repair of the tracheal injury. The patient with a rib fracture was treated using long chest straps. The patient with bilateral pneumothorax as well as the patients with cerebral and cervical spine injuries underwent conventional tracheotomy and cervical spine immobilization, and the other injuries were treated by the appropriate specialists.

### Consent (Adult)

We confirm that “Written informed consent was obtained from the patient for the publication of this report and any accompanying images”.

## Results

All 17 patients with closed injury of the cervical trachea due to blunt trauma underwent tracheal exploration, low tracheotomy, and tracheal anastomosis if necessary. If patients also had other injuries, those were treated appropriately. The breathing, phonation, and swallowing functions of 12 patients had returned to normal at 2 weeks after surgery. In these patients, fiberoptic laryngoscopy or bronchoscopy at 2–4 weeks showed that the tracheal injuries had healed well, with no apparent proliferation of granulation tissue. The tracheal tube was removed after 1 month. In one patient with bilateral recurrent laryngeal nerve injury, secondary recurrent laryngeal nerve to phrenic nerve anastomosis was performed, and the tracheal tube was removed after 3 months. Two patients had ongoing hoarseness even though other functions had returned to normal. These patients were able to vocalize a little, but speech was poor. Two patients underwent reoperation: one underwent resection of the stenosis and end-to-end tracheal repair, and the other only had a stent placed in the trachea. In these two patients, respiratory and swallowing functions had recovered at 6 months. All 17 patients were followed up for more than 6 months, and continued to improve.

## Discussion

Closed injury of the cervical trachea due to blunt trauma is uncommon. The complex clinical manifestations and rapid progression of severity present diagnostic and management challenges. The most common symptoms are pain with swallowing, neck pain, varying degrees of dyspnea, cyanosis, cough, hemoptysis, and hoarseness. Physical examination findings include widespread subcutaneous emphysema and skin contusions which rapidly progress to the neck, shoulder, and chest; cyanosis, pneumothorax, vocal cord paralysis, and aphonia. Owing to the serious nature of this injury, the majority of patients die before reaching hospital because of airway compromise. Patients with partial tracheal disruption have a normally positioned trachea and may form a false passage through the traumatized soft tissues, thereby maintaining a low level of ventilation. If there is no major vascular injury, there may be sufficient time to successfully treat the patient. The tracheal injury may not be obvious owing to the lack of an open wound in the neck, and there may not be immediate dyspnea. Some patients initially present with only hemoptysis, hoarseness, and subcutaneous emphysema in the neck, and more obvious features of tracheal injury may be delayed. Symptoms of tracheal injury may be easily overlooked in patients with other serious injuries. As it is difficult to make an early diagnosis, treatment may be delayed, eventually leading to tracheal stenosis or atresia, which may be life-threatening [2]. The rate of early misdiagnosis is reported to be 35–68% [3]. Early diagnosis and treatment are therefore important for ensuring good outcomes, [1] and we need to improve awareness of this injury.

Studies have shown that the following clinical manifestations are highly suspicious of closed injury to the cervical trachea after neck trauma: (1) pain which is exacerbated by swallowing or neck rotation; (2) skin contusions on the neck, dry cough, cyanosis, hemoptysis, subcutaneous emphysema in the neck, and pneumomediastinum; (3) significant worsening of dyspnea when the neck is extended; and (4) hoarseness. It is necessary to assess whether tracheal disruption is partial or complete. Features of partial tracheal rupture include gradually increasing dyspnea, hemoptysis, hoarseness, and subcutaneous emphysema. In complete tracheal rupture, the skin of the anterior neck moves in and out with respiratory activity, and a gap in the tracheal rings can be felt beneath the moving skin. When a closed injury to the cervical trachea is suspected, it is important to perform immediate neck and chest X-rays, or CT scanning including three-dimensional imaging of the trachea [4]. can clearly show the fascial sheaths, artery sheaths, trachea, esophagus, and larynx, and is the most important preoperative diagnostic imaging modality for determining the location, extent, and type of tracheal injury [5-7]. In stable patients, bronchoscopy can be used to determine the precise location and degree of tracheal injury.

The main therapeutic aim is to maintain the patency of the airway. If necessary, emergency intubation or tracheotomy should be performed [8,9]. Hemostasis should be achieved, and vital signs should be stabilized. As soon as possible, the patient should also be assessed and treated for hemorrhagic shock, traumatic brain injury, and injury to other important organs. Early repair of injuries to the trachea, pharynx, larynx, and other airways helps to preserve the function of these structures [10].

There is controversy regarding whether airway patency is best maintained by tracheal intubation or by tracheotomy [1,11]. We advocate not using either tracheotomy or blind oral or nasal intubation if the diagnosis has not been confirmed. Blind oral or nasal intubation in patients with tracheal injury can lead to false extratracheal intubation and irreversible damage to the airway. If tracheotomy is performed before confirmation of the diagnosis, the distal end of the disrupted trachea may retract into the thoracic cavity, causing airway obstruction and death. After confirmation of the diagnosis, we advocate that patients with significant tracheal injury should undergo standard tracheostomy to reduce the risk of potentially serious damage to the airway. In patients with partial tracheal rupture, orotracheal intubation may aggravate the damage and lead to massive hemorrhage, causing worsening of dyspnea [12]. Intubation under anesthesia should be performed by a skilled operator, preferably with fiberoptic guidance, using a relatively small endotracheal tube. If the initial intubation attempt is unsuccessful, immediate tracheotomy should be performed. In patients with complete tracheal disruption, both the proximal and distal ends may retract. The distal end of the trachea may retract into the thoracic cavity, and intubation attempts may increase the separation between the ends of the trachea and cause additional trauma and hemorrhage in the surrounding tissues. If the endotracheal tube cannot be successfully placed in the distal end of the disrupted trachea, respiratory distress may increase and the patient may die owing to airway obstruction. A patient with associated laryngeal injury may have early dyspnea, hemoptysis, and hoarseness, and should undergo immediate tracheotomy.

When closed injury to the cervical trachea due to blunt trauma has been diagnosed, surgical exploration should be performed under anesthetia [13]. A neck incision can be made under local anesthesia, and the strap muscles can be separated to find the distal end of the disrupted trachea and suction any secretions. General anesthesia can then be administered, and bronchoscopy can be used to examine the injury and guide the endotracheal tube into the distal of the trachea [1]. While the patient is under general anesthetic, a transverse incision can be made in the neck to free the tracheal wall while protecting the recurrent laryngeal nerves and blood vessels, and the esophagus can be examined for injury. Tracheal fractures should be repaired as soon as possible, before the formation of surrounding adhesions and scars. Attention should be paid to protecting the tracheal mucosa during treatment, and whenever possible the mucosa should be sutured together to prevent the formation of granulation tissue, scarring, and tracheal stenosis. If there is a large defect in the tracheal mucosa, it is desirable to repair the defect with fascia. If the trachea is completely disrupted, care should be taken to keep the sutures close to trachea, to avoid damaging the recurrent laryngeal nerves and vessels. If the tracheal cartilage rings are broken in multiple places, they should be retained as much as possible to support the repair. If the cartilage fragments cannot be retained, two or three cartilage rings can be removed, the distal end of the trachea can be freed, and the larynx can be moved downwards, allowing the ends of the trachea to be sutured back together. The suture line can then be reinforced using the adjacent tissues. Cartilage fragments measuring <5 cm should be removed if possible, to avoid interference with the anastomosis. The ends of the trachea are anastomosed by suturing the cartilage rings and the mucosal layers, and then suturing the tracheal fascia for reinforcement. Large defects in the trachea should be repaired using stainless steel wire mesh.

There is controversy regarding whether to retain the endotracheal tube after repair of a tracheal injury. Some surgeons believe that the tube should be retained to prevent airway obstruction, whereas others believe that retaining the tube can cause softening of the C-shaped cartilaginous rings or proliferation of intratracheal granulation tissue [14-16]. We believe that the endotracheal tube should be retained in patients have undergone repair of severe injuries to the larynx or trachea. In patients without laryngeal trauma, who have undergone satisfactory repair of their tracheal injury, the endotracheal tube need not be retained. In these patients only a nasotracheal tube is required, which can be removed after 48 hours. There is no consensus regarding the ideal time of removal of an anastomotic stent. Some studies have shown that removal of the stent at 4 weeks after surgery is feasible. It has been reported that an endotracheal tube can be used instead of a stent for a week. Patients who had medical gloves filled with iodinated gauze or water placed in the trachea for 3 weeks did not have tracheal stenosis after 6 months of follow-up [17].

Postoperatively, the head should be kept forward to reduce anastomotic tension. Patients should receive intensive airway care to prevent infection. Antitussive medication should be administered to avoid severe cough that would induce a rise in airway pressure. Corticosteroid administration reduces inflammation and inhibits proliferation of granulation tissue, which helps to prevent postoperative tracheal stenosis caused by the anastomotic scar.

In this study, 12 of the 17 patients recovered normal breathing, phonation, and swallowing functions, with good healing of the tracheal injury and no significant granulation tissue, at 2–4 weeks after surgery. In one case with bilateral recurrent laryngeal nerve injury, secondary recurrent laryngeal nerve to phrenic nerve anastomosis was performed. In two other patients with residual hoarseness, respiratory and swallowing functions returned to normal, but speech remained poor. Two patients required reoperation, including one who only had a stent placed. In this patient, the stent was removed after 6 months, and there was good recovery of respiratory and swallowing functions.

## Conclusions

**Figure 5:**
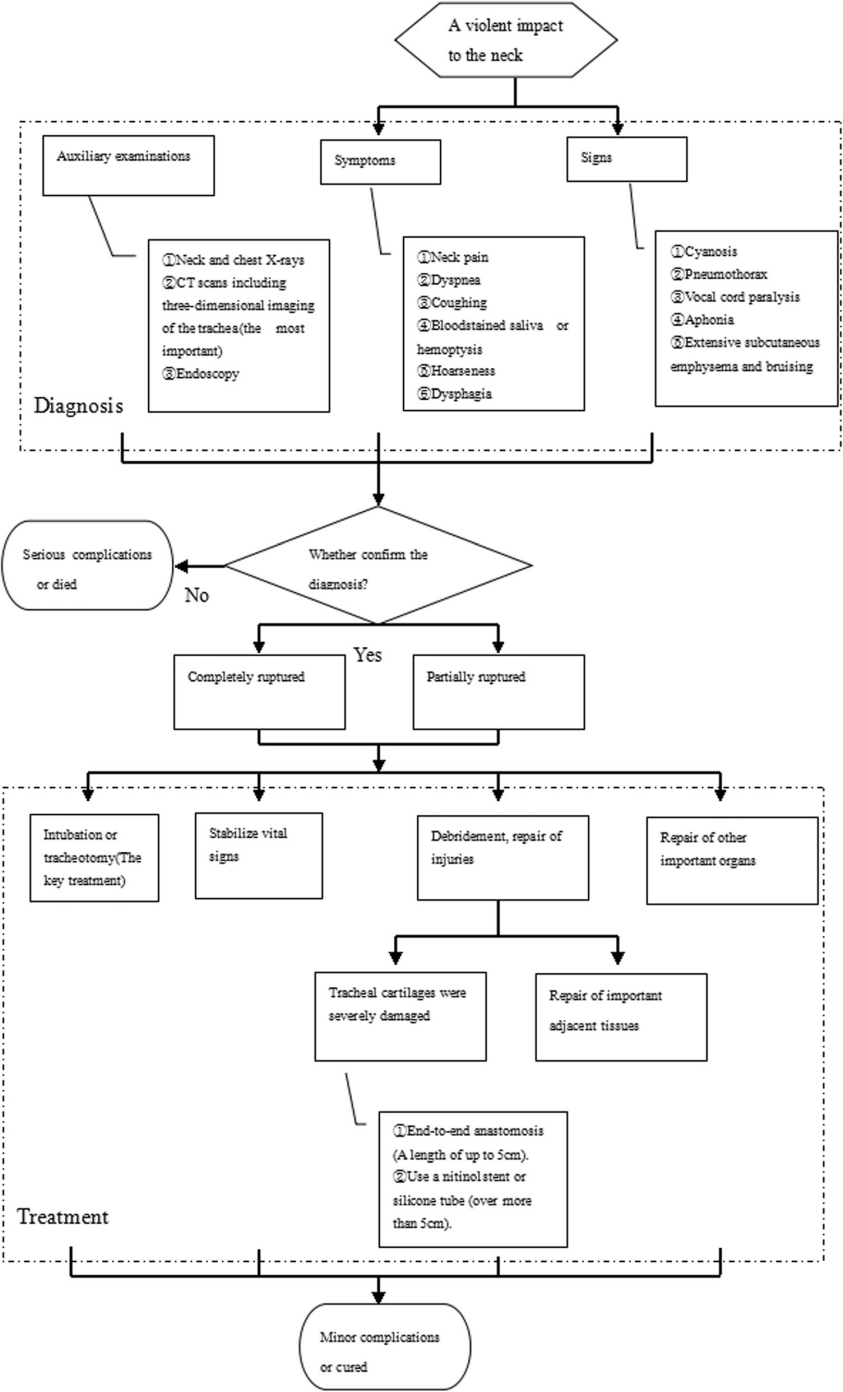
The algorithm of diagnosis and treatment of closed injury of the cervical trachea due to blunt trauma.

Closed injury of the cervical trachea due to blunt trauma is rare and presents diagnostic and management challenges. Early diagnosis is important, and a high threshold of suspicion is required, especially in cases of head, neck, or chest trauma, and in patients with incongruous clinical findings who respond poorly to therapeutic measures. We strongly advise CT scan or endoscopy as reliable diagnostic modalities for closed injury to the cervical trachea. Early recognition and emergent referral for definitive airway management are important [8] (Figure 5).

## Competing interests

There are no competing interests associated with this article.

## Authors’ contributions

DY designed the study, performed the surgery,acquired of data,conducted the data analysis and drafted the manuscript. ZS performed the surgery, helped in the data collection and revised the manuscript. YZ performed the surgery, helped in the data analysis and revised the manuscript. SQ and CK co-designed the study, organized the data collection and helped to draft the manuscript. All authors read and approved the final manuscript.
